# A Second Appeal to the People of Pennsylvania on the Subject of an Asylum for the Insane Poor

**Published:** 1840-10-24

**Authors:** 


### BIBLIOGRAPHICAL NOTICE.

*A second appeal to the People of Pennsylvania on the subject of an Asylum for the Insane Poor.*

WHEN the movement in behalf of this object was originally made, we expressed our sense of its importance as well as our confident hopes of its success. These hopes, for a time suspended, from economical considerations on the part of the Governor of the State, we trust may be realized at the coming session of the Legislature. The enlightened philanthropists with whom the matter originated, are renewing their praise-worthy exertions, and offer in the pamphlet before us, a second able appeal to the people and authorities of Pennsylvania. A startling picture is presented of the extent of indigent, unrelieved insanity within the commonwealth, while the practicability of establishing and maintaining an Asylum at a very moderate expenditure is clearly made out. The appeal is from the pen of Dr. Dunglison.

